# Random forest prediction of Alzheimer’s disease using pairwise selection from time series data

**DOI:** 10.1371/journal.pone.0211558

**Published:** 2019-02-14

**Authors:** P. J. Moore, T. J. Lyons, J. Gallacher, for the Alzheimer’s Disease Neuroimaging Initiative

**Affiliations:** 1 Mathematical Institute, University of Oxford, Oxford, United Kingdom; 2 Department of Psychiatry, University of Oxford, Oxford, United Kingdom; Nathan S Kline Institute, UNITED STATES

## Abstract

Time-dependent data collected in studies of Alzheimer’s disease usually has missing and irregularly sampled data points. For this reason time series methods which assume regular sampling cannot be applied directly to the data without a pre-processing step. In this paper we use a random forest to learn the relationship between pairs of data points at different time separations. The input vector is a summary of the time series history and it includes both demographic and non-time varying variables such as genetic data. To test the method we use data from the TADPOLE grand challenge, an initiative which aims to predict the evolution of subjects at risk of Alzheimer’s disease using demographic, physical and cognitive input data. The task is to predict diagnosis, ADAS-13 score and normalised ventricles volume. While the competition proceeds, forecasting methods may be compared using a leaderboard dataset selected from the Alzheimer’s Disease Neuroimaging Initiative (ADNI) and with standard metrics for measuring accuracy. For diagnosis, we find an mAUC of 0.82, and a classification accuracy of 0.73 compared with a benchmark SVM predictor which gives mAUC = 0.62 and BCA = 0.52. The results show that the method is effective and comparable with other methods.

## Introduction

Alzheimer’s disease (AD) is an irreversible brain disorder which progressively affects cognition and behaviour, and results in an impairment in the ability to perform daily activities. It is the most common form of dementia in older people affecting about 6% of the population aged over 65, and it increases in incidence with age. The initial stage of AD is characterised by memory loss, and this is the usual presenting symptom. Memory loss is one constituent of mild cognitive impairment (MCI) which can be an early sign of Alzheimer’s disease. MCI is diagnosed by complaints of subjective memory loss (preferably corroborated by a close associate or partner of the individual), impairment of memory function, unimpaired general cognition and behaviour but with no evidence of dementia [1]. MCI does not always progress to dementia or to a diagnosis of Alzheimer’s disease, but those with amnestic mild MCI, the type of MCI characterised by memory impairment, are more likely to develop dementia than those without this diagnosis. In cases where an individual does develop Alzheimer’s disease, the phase of MCI ends with a marked decline in cognitive function lasting two to five years in which semantic memory (the recall of facts and general knowledge) and implicit memory (the long-term, nonconscious memory evidenced by priming effects) also becomes degraded.

Clinical diagnosis of dementia relies on information from a close associate or partner of the individual, and on cognitive and physical examinations. Once dementia is diagnosed it is usually subclassified into Alzheimer’s disease, vascular dementia or Lewy Body dementia [2, 3], these three classes making up the majority of cases. Risk factors for Alzheimer’s disease are multifarious, including sociodemographic (in particular age), genetic (notably ApoE status), and medical history (such as a diagnosis of depression). The cause of Alzheimer’s disease is not fully understood, but plaques containing amyloid *β*–peptide (A*β*) in brain tissue and neurofibrillary tangles containing tau protein are the primary histological features [4].

### Predicting Alzheimer’s disease

The disease leads to an progressive, irreversible loss of brain function, so prospective drug therapies need to be tested for efficacy as early in the process as possible. As a result there is a demand for the timely prediction of diagnosis for testing therapies which might inhibit or prevent tissue damage. There has been much research effort put into the prediction of an AD diagnosis among those who are diagnosed with MCI, in particular using imaging to detect early signs of the disease pathology: a meta analysis of 32 structural MRI or amyloid PET imaging studies that reported conversion to AD in patients with MCI is given by Seo *et al*. [5]. This analysis concluded that amyloid PET is a better predictor of progression to AD than MRI atrophy measures (effect size 1.32 vs 0.77), but that MRI on entorhinal cortex atrophy (effect size 1.26) is comparable in prediction value to that of amyloid PET. Another comparison of biomarker predictivity found that the highest predictive accuracy was achieved by combinations of amyloidosis and neurodegeneration biomarkers [6]. The individual biomarker with the best performance was [18F]-fluorodeoxyglucose-positron emission tomography (FDG-PET) which measures temporoparietal hypometabolism.

The application of machine learning to predicting Alzheimer’s disease from neuroimaging data is an active area of research. A review by Weiner *et al*. [7] lists 49 papers which use the ADNI data, with a summary of input data, feature selection and prediction methods. Of these methods the majority used MRI data, sometimes supplemented with other neuroimaging data (usually PET) and with other modes such as cognitive, demographic and genetic data. Most studies used the support vector machine (SVM) as the learning method, three papers used random forests [8–10], and the remaining studies used score-based or other classification approaches. The tasks attempted were usually to distinguish diagnostic categories NL (healthy), MCI, and AD to forecast conversion, usually from MCI to AD. For example, Moradi *et al*. [11] used features from MRI images, age and cognitive measures to predict MCI to AD conversion from one to three years before diagnosis was made. They used an SVM classifier to distinguish patients who remained as MCI over at least 3 years (sMCI) from those whose diagnosis was MCI at baseline and who converted to AD within 3 years (pMCI). Using MRI features they achieved a 10-fold cross-validated AUC score of 0.77 in discriminating pMCI from sMCI patients; their aggregate biomarker based on MRI data together with baseline cognitive measurements and age gave an AUC score of 0.90.

A review of the use of random forests classifying of neuroimaging data in Alzheimer’s disease is given by Sarica *et al*. [12]. They examined 12 studies, 10 of which used ADNI data, and the majority used features derived from MRI imaging data. Across the studies they found an accuracy of about 90% for classification of AD *vs*. HC (healthy controls) and around 82% for the classification of MCI *vs*. HC and stable MCI *vs*. progressive MCI. Three studies which reported a multiclass classification of AD *vs*. MCI *vs*. HC had results ranging from 53% to 96%. Similar results for multiclass classification are reported by Dimitriadis *et al*. who also include a review of machine learning techniques applied to neuroimaging data [13]. Other reviews of diagnostic classification using neuroimaging data are provided by Rathore *et al*. [14], Arbabshirani *et al*. [15] and Falahati *et al*. [16] with the latter two focusing on studies which use MRI images to derive features.

### The TADPOLE grand challenge

One of the challenges of classification in Alzheimer’s disease is the wide range of both data and forecasting tasks, making comparisons across studies difficult or impossible. In this paper we assess the performance of our approach using data from the TADPOLE grand challenge, and we evaluate the results against methods provided by the competition organisers. In the past few years there have been a number of challenges which allow comparison between methods using a common data set and standardised evaluation metrics. The CADDementia challenge [17] compares algorithms for the classification of AD, MCI and controls based on structural MRI data. The top rank in this competition is held by Sørensen *et al*. [18] who looked beyond the usual volumetric biomarkers and used cortical thickness measurements, hippocampal shape and texture, and other measures in addition to volume measurements. Using linear discriminant analysis they achieved an accuracy of 63% on test data. The Kaggle Neuroimaging challenge https://www.kaggle.com/c/mci-prediction [19] is based on the Kaggle machine learning platform and uses data from the Alzheimer’s Disease Neuroimaging Initiative (ADNI), one of the most commonly used data sets for studies of Alzheimer’s disease [7]. The challenge involved a four-fold classification into AD, MCI, MCI converters to AD and controls with pre-processed sets of T1-weighted MRI images as input. The best performing entry to the Kaggle Neuroimaging Challenge used an ensemble of random forest models to achieve an accuracy of 61.9% in a blind external validation dataset [13].

The TADPOLE grand challenge https://tadpole.grand-challenge.org/ is currently taking place with evaluation to be completed by January 2019. The task is a three-fold classification into AD, MCI and control groups, and the prediction of ADAS-13 score and normalised brain volume [20]. The TADPOLE challenge aims to predict the onset of Alzheimer’s disease using different modes of measurement, including demographic, physical and cognitive data. In common with many other studies [7] the data set is derived from ADNI which is comprised of four phases: ADNI-1 (2004), ADNI-GO (2009), ADNI-2 (2011), and ADNI-3 (2016). ADNI-1 registered 200 healthy elderly, 400 participants with MCI, and 200 participants with AD, and the subsequent phases continued to add participants. The main TADPOLE competition involves predicting future data collected as part of the ADNI-3 phase. The organisers also provide a leaderboard dataset separate from the main competition which allows prediction methods from different teams to be evaluated. The results presented in this paper are derived from the leaderboard dataset using a time horizon varying from 1 to 84 months with a test set of 110 participants and 417 prediction points. Since TADPOLE data is based on ADNI, the data used in the preparation of this article were obtained from the (ADNI) database (adni.loni.usc.edu). ADNI is led by Principal Investigator Michael W. Weiner, MD. Up-to-date information can be found at www.adni-info.org.

## Subjects and methods

### Leaderboard data

**Fig 1:**
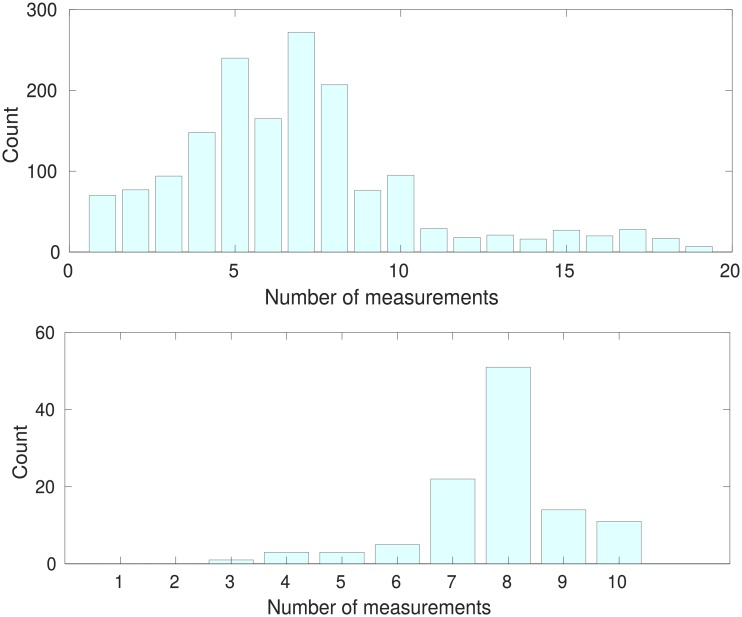
Histograms of time series lengths. Upper: training set LB1 whose time series may cover ADNI, ADNI-GO and ADNI-2. Lower: Set LB2 which is formed only from time series from ADNI-1.

The participants in ADNI have each generated a time series of measurements as monitoring has progressed. The TADPOLE leaderboard dataset consists of three labelled sets of these time series, LB1, LB2 and LB4. The ‘training’ set LB1 has complete time series from 1627 participants. The ‘history’ set LB2 and the leaderboard test set LB4 use time series from a separate group of 110 participants. LB2 has the first part of each time series, from ADNI-1, and LB4 has the remainder from ADNI GO/ADNI-2. Participants in LB2 were not diagnosed with AD at the last ADNI-1 time point. The task is to predict the diagnosis, ADAS-13 score and the normalised ventricles volume for the set LB4 using set LB1 and the participant histories recorded in LB2. The results are evaluated by comparison with LB4 data using a variety of metrics which are described below. No information from LB4 may be used for model training, but demographic and other details about the participants who contributed to LB4 are available from LB2, and past time varying data such as imaging and cognitive measurements are also available from LB2. A histogram of time series lengths for LB1 and LB2 is shown in Fig 1.

Features for prediction are selected from demographic, cognitive and physical data variables. The physical data comprises, among other measurements, MRI data (volumes, cortical thickness, surface area), PET (FDG, AV45 and AV1451), DTI (regional means of standard indices) and levels of markers from cerebral spinal fluid (CSF).

### Evaluation set

For the purposes of training we create an evaluation set whose time series are similar to those in the leaderboard history set LB2. The evaluation set is selected from LB1 by choosing participants matching those in LB2 and whose ADNI-1 time series length is similar. The post-ADNI-1 phases of this matched evaluation set should be similar to that of the test set LB4 and can be used to assess prediction accuracy during training. To create the evaluation set we examine each participant time series in LB2 and find those participants in LB1 who have a matching gender, ApoE4 status and age (to within 5 years) and whose diagnosis matches at the start and end of the ADNI-1 phase. If more than one matching participant is found we select the one with the closest match for the time series length in the ADNI-1 phase. The demographic characteristics and ApoE4 status of the participants from set LB2 and the matched evaluation set are shown in Table 1.

**Table 1:** Demographic characteristics and ApoE4 status for participants from the set LB2 compared with a matched evaluation set selected from LB1. The rows are the sample size *n*, age as minimum, mean and maximum, gender and ApoE4 status.

	*LB2*	*Evaluation set*
*n*:	110	92
*Age*:	59.9 (75.1) 87.9	57.8 (75.3) 84.8
*Male*:	60.9%	60.9%
*Female*:	39.1%	39.1%
*APOE4 0*:	70.0%	68.5%
*APOE4 1*:	27.3%	29.4%
*APOE4 2*:	2.7%	2.2%

To select features and set algorithm parameters we minimize the cross-validation error using a training set. The training set comprises all the time series from LB1 (including points from all three ADNI phases, ADNI-1 ADNI-GO and ADNI-2) and the ADNI-1 points from the evaluation set. The time points from the evaluation set after the ADNI-1 phase are not included in the training set.

### Forecasting method

The purpose of a forecasting method is to predict a participant’s condition at points in the future using demographic, cognitive and physical data variables from time points in the participant’s history. A common approach in time series prediction is to use weighted combinations of past data points to predict the next data point. Time series models in general encode a mapping from an input space to the output, where time is not one of the input dimensions. However, many time series in the training data are short and the sampling periods are irregular so much of the information in the training data lies in the mapping from the time delay between measurements to the output rather than in the sequence of input values. Irregular sampling and missing data can be managed by interpolation or by using appropriate methods such as Gaussian process regression [21], but these approaches entail making assumptions about the distributions. Another approach is to use an input space formed of demographic variables λ, last diagnosis *g*_(*t*−Δ*t*)_ and time since last measurement Δ*t*, and map vectors from this space to the output variable *g*_*t*_. Assuming additive error, the model is,

$$\begin{array}{c} g_{t}=f\left(\lambda ,\;g_{\left(t-\Delta t\right)}\;,\Delta_{t}\right)+\epsilon_{t} \end{array}$$ (1)

As noted above, most studies on classifying neuroimage data for Alzheimer’s disease use SVMs, although random forests are also represented [7]. Random forests work well with a mixture of quantitative and categorical features and unlike SVMs they handle multiple output classes natively. They are easy to train and they provide a variable importance measure. These considerations have informed our choice of a random forest for this study.

**Fig 2:**
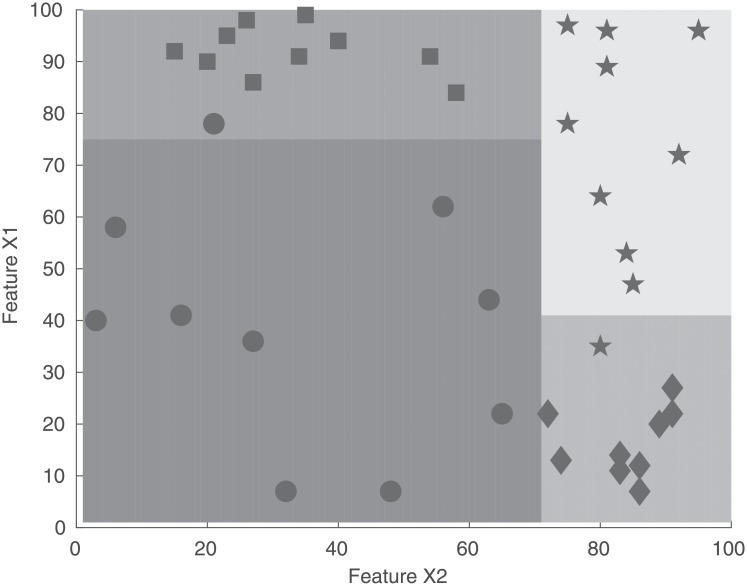
Partition of a 2D input space using two features, X1 and X2. Feature X2 is first used to bisect the square, then each rectangle is bisected using feature X1. The different marker styles denote unique classes.

A random forest is formed from an ensemble of decision trees where each tree partitions the input space into a set of rectangles to minimize the loss function [22]. The training process for an individual tree iterates over all the variables and selects the variable and split point that gives the best partition for the training data. The best partition is that that which gives the minimum total impurity in the two subsets that are formed. The process is repeated until a stopping criterion (such as a minimum number of points in the rectangle) is met. Fig 2 illustrates the partition algorithm in the case of two input variables.

To reduce the variance of the final estimate the random forest algorithm randomly selects *sqrt*(*n*) of the *n* features at every split point [23]. The overall estimate for regression is the average over the ensemble of estimators, and for classification it is the majority class. The algorithm parameters are the number of trees in the ensemble and the minimum number of data points on a leaf. These values are found by minimizing the out-of-bag (OOB) prediction error for each parameter value, where the OOB prediction is found by classifying observations using trees grown without using those observations. More detail about the algorithm and the theory of random forests is given in Hastie *et al*. [22].

### Reference methods

For comparison with our random forest model we show results for a linear mixed effects model and a benchmark support vector machine (SVM), both provided by the competition organisers. The linear mixed effects model uses two fixed parameters (intercept and slope), one random effect (intercept) and one covariate for the slope, APOE. The SVM uses two features: ADAS13 and Ventricles divided by ICV_bl. For diagnosis it uses a linear support vector machine classifier with diagnosis at the next follow-up as the label. For ADAS13 and Ventricles prediction it uses linear support vector regression with the value at the next follow-up as the label.

### Metrics

Forecasts for each diagnosis are made as probabilities for each of the classes, NL, MCI and AD. We report the multiclass area under the receiver operating curve (mAUC) and the balanced classification accuracy (BCA). The mAUC is based on Hand and Till’s extension of AUC to multiple classes [24] which takes the average AUC over all the pairs of classes, where each pair of AUCs $\hat{A}\left(c_{i},c_{j}\right),\hat{A}\left(c_{j},c_{i}\right)$ is itself averaged. The balanced classification accuracy (BCA) is the mean of the true positive rate and true negative rate. Forecasts for the quantitative outputs ADAS-13 and VENTS-ICV are evaluated using the mean absolute error (MAE). These measures are used by the TADPOLE competition, and the organisers have shared the codes for their computation. We reimplemented the codes and verified the results by comparison with the competition leaderboard.

## Results and discussion

We trained the random forest only on time series with at least 4 points, this minimum having been determined during training. The optimum value for the number of trees was 60, and for the minimum number of data points in a leaf it was 5. The average cross-validation accuracy for the evaluation set for diagnosis was AUC = 0.82 (SD 0.09). For ADAS-13 prediction the error was MAE = 5.56 (0.58) and for VENTS-ICV, MAE = 0.0021 (0.001).

### Features

The variables selected during training are shown in Table 2. These were chosen by starting from a prior set of variables RID, TIME_DELAY, DX, AGE, GENDER, MMSE and adding variables to increase the prediction accuracy. The set of features was optimised for diagnosis as the output variable with two variables added for predicting ADAS-13: the input ADAS13 and its slope ΔADAS13. By contrast, the feature set for the VENTS output was TIME_DELAY, VENTRICLES and ΔVENTRICLES, and for ICV it was TIME_DELAY, ICV and ΔICV. The output VENTS-ICV is the ratio of these two predictions.

**Table 2:** The set of variables from which features are selected for prediction. The variable MMSE is binary and found by thresholding the raw value at 26. The output VENTS-ICV is the ratio of VENTS and ICV which are predicted using separate models. VENTS is predicted using VENTRICLES, ΔVENTRICLES and TIME_DELAY. ICV is predicted using ICV, ΔICV and TIME_DELAY.

Variable	Meaning	Diag	ADAS-13	VENTS-ICV
DX	Diagnosis (NL or MCI or AD)	✓	✓	
AGE	Age	✓	✓	
GENDER	Gender	✓	✓	
APOE	ApoE4 status	✓	✓	
MMSE	MMSE Mini-mental state examination	✓	✓	
CDRSB	CDRSB	✓	✓	
FAQ	Functional activities questionnaire	✓	✓	
RID	Participant identifier	✓	✓	
MIDTEMP	Middle temporal gyrus	✓	✓	
TIME_DELAY	Number of months delay	✓	✓	✓
ADAS13	Alzheimer’s Disease Assessment Scale		✓	
ICV	Intracranial volume			✓
VENTRICLES	Ventricles volume			✓
ΔMMSE	MMSE slope	✓	✓	
ΔADAS13	Alzheimer’s Disease Assessment Scale slope		✓	
ΔHIPPOCAMPUS	Hippocampus volume slope	✓	✓	
ΔVENTRICLES	Ventricles volume slope	✓	✓	✓
ΔICV	Intracranial volume slope			✓

**Fig 3:**
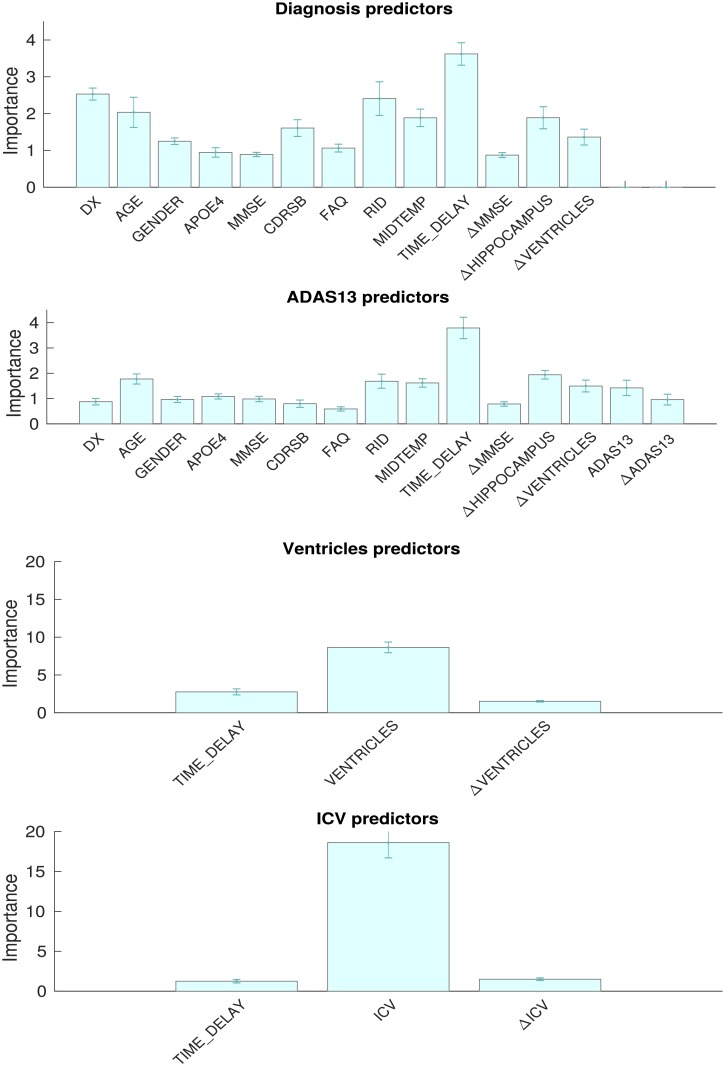
Variable importance for diagnosis, ADAS-13, ventricles and intracranial volume prediction.

The relative importance of these features is shown in Fig 3. Importance is measured by the increase in prediction error when the values of the variable are permuted across the out-of-bag observations. This measure is computed for every tree, then averaged over the ensemble and divided by the standard deviation. For predicting diagnosis, the initial diagnosis DX, participant identifier RID and the time delay TIME_DELAY are the three most important. This finding shows that the method is working as expected: the best predictor of future diagnosis for an individual will be the last diagnosis, especially for short time horizons, and time will be the greatest determinant of change in that diagnosis. The participant identifier carries more diagnostic information specific to an individual in addition to the last diagnosis. For predicting the quantitative output ADAS-13, TIME_DELAY is again highly predictive, while the diagnosis DX is not. Otherwise the profile of the two outputs is similar with AGE, RID, MIDTEMP and ΔHIPPOCAMPUS important in both cases. For predicting both the quantitative outputs VENTS and ICV, from which the target VENTS-ICV is found, the most important predictor is the variable itself, with ΔTIME_DELAY and the slope being used to adjust this last value prediction.

### Test set results

The confusion matrix for diagnosis prediction is shown in Table 3. The matrix summarises a total of 417 test points for 110 participants in the test set LB4. For predicting NL diagnoses, the accuracy is 99%, for MCI, 59% and for AD, 29% giving an overall accuracy of 72%. The classifier identifies NL diagnoses accurately but misclassifies about a third of MCI diagnoses as NL, and more than half of AD diagnoses as MCI. This appears to be a poor performance in identifying MCI and AD diagnoses. However in order to score on the accuracy measure the time of transition from one diagnostic state to another (NL to MCI etc.) has to be estimated within some margin of error over a period of up to 7 years.

**Table 3:** Confusion matrix for predicting 417 diagnosis points of the 110 participants in test set LB4, where the forecast horizon is up to 7 years. The labels are as follows, NL: healthy, MCI: mild cognitive impairment, AD: Alzheimer’s disease. The overall accuracy is 0.72.

	*Predicted diagnosis*					
		NL	MCI	AD	Total	Accuracy
*Actual diagnosis*	NL	193	2	0	195	0.99
	MCI	54	88	8	150	0.59
	AD	6	45	21	72	0.29

Table 4 shows the test set metric values for diagnosis, ADAS-13 and VENTS-ICV prediction. For diagnosis prediction the random forest has mean accuracy measures of mAUC = 0.80 and BCA = 0.74, outperforming both the linear mixed effects model and SVM references. For ADAS-13 prediction the error for the random forest model is MAE = 5.24, compared with the mixed effects model of MAE = 5.87 and the benchmark SVM of MAE = 8.13. For VENTS-ICV prediction the mean test set error for the random forest model is MAE = 0.0026, compared with the mixed effects model of MAE = 0.0034 and the SVM of MAE = 0.0098.

**Table 4:** Test set results for the random forest, mixed effect and SVM estimators. The values are the mean over ten splits of the test data with the standard deviation in brackets.

Method	Diagnosis		ADAS-13	VENTS-ICV
	mAUC	BCA	MAE	MAE
Random forest	0.80 (0.06)	0.74 (0.05)	5.24 (1.39)	0.0026 (0.00130)
Mixed effects	0.77 (0.07)	0.68 (0.07)	5.87 (1.26)	0.0034 (0.00098)
SVM	0.62 (0.07)	0.51 (0.04)	8.13 (1.02)	0.0098 (0.00078)

In the section on Predicting Alzheimer’s disease we saw that the reviews by Sarica *et al*. [12] and Dimitriadis *et al*. [13] found accuracies ranging from 53% to 96% for multiclass classification of diagnosis. By comparison we found an accuracy of 74% for predicting diagnosis, as shown in Table 4. However, although our study uses data that is derived from ADNI, both the data and forecasting task are different from previous studies. In our case, the test data has been selected specifically for the TADPOLE competition leaderboard, and the task is to predict diagnosis over a forecast horizon which can vary between one month and 7 years: other studies generally predict a transition over a fixed period. For a meaningful comparison methods have to be compared with others from the same competition, as shown in the leaderboard table reproduced in Table 5. The leaderboard shows entries in rank order where the rank is determined by the lowest sum of individual ranks for mAUC, ADAS-13 MAE and VENTS-ICV MAE. Two additional metrics are computed for the leader board: the weighted error score (WES) and the coverage probability accuracy (CPA). The weighted error score is the absolute error weighted by the inverse of the confidence interval range. The coverage probability accuracy is defined as, *CPA* = |*j* − 0.5|, where *j* is the proportion of measurements falling within the 50% confidence interval. The original table can be found at https://tadpole.grand-challenge.org/leaderboard from which it can be seen that only 6 of 17 teams (with repeated submissions) appear to outperform the method described in this paper.

**Table 5:** Competition leaderboard table at 4 May 2018 where each row represents an entry from a competition team listed in rank order. The first highlighted row shows the results for our random forest estimator, the middle highlighted entry shows the results for a linear mixed effects model and the bottom highlighted entry for a support vector machine model. There are three target outcomes for prediction: 1) Diagnosis, 2) the ADAS-13 score, and 3) VENTS-ICV which is the ventricles volume divided by intracranial volume. The overall rank is determined by the lowest sum of ranks from mAUC, ADAS-13 MAE and VENTS-ICV MAE.

	Diagnosis		ADAS-13			VENTS-ICV		
	mAUC	BCA	MAE	WES	CPA	MAE	WES	CPA
	0.91	0.83	3.62	3.62	0.11	0.0020	0.0018	0.13
	0.93	0.85	3.72	3.10	0.02	0.0020	0.0016	0.15
	0.93	0.85	3.72	3.10	0.02	0.0020	0.0016	0.15
	0.91	0.83	3.67	3.67	0.12	0.0024	0.0022	0.08
	0.91	0.74	3.73	3.70	0.01	0.0028	0.0023	0.32
	0.89	0.78	4.16	4.16	0.39	0.0023	0.0023	0.47
	0.89	0.82	3.76	3.76	0.12	0.0034	0.0029	0.15
	0.89	0.82	3.80	3.80	0.11	0.0034	0.0029	0.14
	0.87	0.78	4.12	4.08	0.03	0.0027	0.0027	0.01
	0.87	0.69	4.41	4.41	0.30	0.0026	0.0026	0.46
	0.84	0.74	4.54	4.17	0.49	0.0025	0.0021	0.49
	0.89	0.81	3.81	3.81	0.11	0.0057	0.0041	0.01
	0.88	0.80	3.87	3.87	0.11	0.0049	0.0038	0.05
	0.91	0.74	3.73	3.70	0.01	0.0092	0.0092	0.01
	0.80	0.74	4.51	4.49	0.40	0.0027	0.0027	0.25
Random forest	0.82	0.73	5.19	4.57	0.07	0.0023	0.0019	0.11
	0.76	0.67	4.34	4.30	0.08	0.0022	0.0021	0.08
	0.88	0.80	5.00	4.78	0.03	0.0030	0.0030	0.05
	0.88	0.80	3.92	3.92	0.10	0.0060	0.0043	0.01
	0.86	0.70	4.56	3.69	0.14	0.0034	0.0032	0.43
	0.81	0.73	5.13	5.14	0.01	0.0027	0.0028	0.20
	0.81	0.73	4.09	4.09	0.09	0.0045	0.0038	0.01
	0.80	0.74	4.51	4.49	0.40	0.0038	0.0038	0.42
	0.80	0.68	4.14	4.14	0.29	0.0040	0.0040	0.38
	0.80	0.66	4.81	4.81	0.21	0.0038	0.0038	0.10
	0.80	0.74	4.60	4.60	0.35	0.0041	0.0041	0.12
	0.88	0.69	4.98	4.98	0.34	0.0066	0.0066	0.27
	0.78	0.71	4.60	4.60	0.35	0.0041	0.0041	0.12
	0.79	0.69	6.68	5.54	0.05	0.0028	0.0023	0.32
	0.81	0.72	4.70	4.70	0.09	0.0070	0.0070	0.03
	0.77	0.65	4.83	4.83	0.20	0.0038	0.0038	0.07
	0.87	0.70	4.91	4.79	0.36	0.0073	0.0073	0.46
Mixed effects	0.77	0.68	5.85	5.85	0.38	0.0032	0.0032	0.34
	0.71	0.63	6.37	6.71	0.39	0.0026	0.0026	0.32
	0.71	0.63	6.37	6.74	0.25	0.0026	0.0026	0.27
	0.79	0.66	4.69	4.69	0.09	0.0093	0.0093	0.01
	0.76	0.69	5.00	4.98	0.35	0.0042	0.0042	0.38
	0.72	0.62	5.70	5.70	0.41	0.0036	0.0036	0.43
	0.73	0.59	9.63	9.63	0.45	0.0029	0.0029	0.48
	0.80	0.68	6.00	6.00	0.11	0.0075	0.0075	0.17
	0.71	0.58	9.70	9.70	0.40	0.0029	0.0029	0.26
	0.74	0.68	5.70	4.60	0.21	0.0070	0.0042	0.35
	0.74	0.68	5.70	4.60	0.21	0.0070	0.0042	0.35
	0.77	0.65	6.73	6.73	0.13	0.0094	0.0094	0.02
	0.78	0.68	7.39	7.39	0.12	0.0095	0.0095	0.04
	0.78	0.66	8.43	5.09	0.48	0.0096	0.0095	0.50
				…				
SVM	0.62	0.52	8.11	8.11	0.50	0.0098	0.0098	0.50

### Discussion

We have shown how a machine learning method can learn the relationship between pairs of data points at different time separations for the prediction of Alzheimer’s disease. The advantage of this scheme is that it can easily be applied to data with irregularly sampled or missing data points. It outperforms linear mixed effects and SVM methods. A limitation of the approach is the direct comparison with just two methods both with a small number of predictors. However the comparison against other methods in the leaderboard demonstrates the effectiveness of the pairwise prediction method, and shows that it performs well against a range of other approaches. The results also show that random forests are an effective choice for this kind of classification challenge. They take both quantitative and qualitative input variables, they are easy to train and they perform well over a wide range of applications.

A possible application for predicting diagnosis is the application to clinical trials. In selecting participants for clinical trials, a positive PET scan is commonly used as part of the inclusion criteria. However PET imaging is expensive, so when a positive scan is one of the trial inclusion criteria it is desirable to avoid screening failures. One possibility is to preselect candidates using machine learning before applying the trial criteria. The variables and methods found from the TADPOLE competition could be used to inform a clinical trials simulation. Even relatively modest values for sensitivity and specificity for predicting a diagnosis of Alzheimer’s disease might have major resource savings for trials. Further work remains to determine prediction variables within the constraints of available variables and the period of monitoring.

In this paper we have described a prediction method that makes no assumptions about the dynamics of input time series, and it is applicable to irregularly sampled data of any length. The results are better than the competition benchmark and they validate the method as effective. We look forward to the final results of the TADPOLE competition which will provide a definitive comparison of the different prediction methods and features.
